# Precision Genomic Practice in Oncology: Pharmacist Role and Experience in an Ambulatory Care Clinic

**DOI:** 10.3390/pharmacy8010032

**Published:** 2020-03-08

**Authors:** Farah Raheem, Pauline Kim, Meagan Grove, Patrick J. Kiel

**Affiliations:** Indiana University Health Simon Cancer Center, Indianapolis, IN 46202, USA; fraheem@iuhealth.org (F.R.); pkim3@iuhealth.org (P.K.); Patrick.Kiel@gmail.com (P.J.K.)

**Keywords:** precision genomics, precision medicine, precision oncology, targeted therapy, oncology pharmacist, molecular tumor board, next generation sequencing

## Abstract

Recent advancements in molecular testing, the availability of cost-effective technology, and novel approaches to clinical trial design have facilitated the implementation of tumor genome sequencing into standard of care oncology practices. Current models of precision oncology practice include specialized clinics or consultation services based on a molecular tumor board (MTB) approach. MTBs are comprised of interprofessional teams of clinicians and scientists who evaluate tumors at the molecular level to guide patient-specific targeted therapy. The practice of precision oncology utilizing MTB-based models is an emerging approach, transforming precision genomics from a novel concept into clinical practice. This rapid shift in practice from cytotoxic therapy to targeted medicine poses challenges, yet brings exciting opportunities to clinical pharmacists practicing in hematology and oncology. Only a few precision genomics programs in the United States have a strong pharmacy presence with oncology pharmacists serving in leadership roles in research, interpreting genomic sequencing, making treatment recommendations, and facilitating off-label drug procurement. This article describes the experience of the precision medicine clinic at the Indiana University Health Simon Cancer Center, with emphasis on the role of the pharmacist in the precision oncology initiative.

## 1. Introduction

Translation of genetic testing into standard clinical practice in oncology was pioneered by scientific and clinical efforts in areas related to pharmacogenetics and pharmacodynamics [1]. Recent advancements in molecular testing, availability of cost-effective technology, and novel approaches to clinical trial design have facilitated the implementation of tumor genome sequencing into standard of care oncology practices [2]. Additionally, interrogating tumor genomes has become especially valuable in an unprecedented era of a rapidly evolving market of targeted drug discovery [3]. Tumor genomic variants can be classified into germline or somatic. Germline genetics are defined as inherited variation from gametes and are implicated in hereditary diseases, including cancer [4]. Germline mutations in BRCA1 and BRCA2 have been shown to be associated with increased risk of developing breast, ovarian, prostate, and other malignancies [5,6]. Somatic mutations, such as EGFR and KRAS mutations, are acquired genetic variations in the tumor and can be utilized as prognostic and predictive biomarkers [7,8,9,10,11]. 

Identification of somatic mutations as oncogenic drivers of malignancy has revolutionized the treatment of various malignancies such as chronic myeloid leukemia and non-small lung cancer [11]. We owe this paradigm shift in targeted malignancy treatment to innovations in biotechnology and genetic testing platforms, which greatly contributed to our understanding of tumor molecular pathogenesis. Historically, Sanger sequencing methods were used to identify mutations in cancer genomes. These first-generation sequencing platforms are limited to detect short sequences of DNA with low sensitivity, thus whole-genome sequencing was costly and inefficient with these methods. Whole-genome sequencing allows identification of all point mutations and structural rearrangements in both somatic and germline tissues [12]. On the other hand, next generation sequencing (NGS), also known as massively parallel sequencing, is a high-throughput testing platform used to detect multiple types of genetic alterations including single nucleotide polymorphisms, deletions, insertions, copy number alterations, and rearrangements [13,14]. On average, NGS technology has the capability of analyzing 200 to 592 genes with full exon coverage including point mutations, indels, and copy number alterations [13]. Today, most testing panels utilize NGS technology. Foundation Medicine (There are several other NGS tests available from companies such as Paradigm, Illumina/Solexa, Life/APG, TransMed, and Helicos BioSciences.) was the first to receive FDA approval in November 2017 as a companion based diagnostic test for certain chemotherapy agents, such as LYNPARZA^®^ (Olaparib) for first-line maintenance therapy in BRCA-mutated advanced ovarian cancer [12,15,16]. Biotechnology has not only facilitated our understanding of cancer at the genetic and molecular level, but it has also offered an extensive amount of information that requires interpretation by an organized multidisciplinary team of scientists and healthcare providers for application to clinical practice.

The Precision Medicine Initiative, also known as the Cancer Moonshot Initiative, was established by President Barack Obama and led by Vice President Joe Biden in 2016. This initiative was launched with a $215 million investment to pioneer a new model of patient-powered research led by the National Institutes of Health (NIH) and $70 million dedicated to the National Cancer Institute (NCI), to scale up efforts to identify genomic drivers in cancer and to develop more effective approaches to cancer treatment [17]. Currently, the NCI is leading the Molecular Analysis for Therapy Choice (NCI-MATCH) trial which employs a basket trial design (Basket trial is a type of clinical trial design, which aims to enroll patients with different types of cancers or tumor histology and assign targeted treatment based on specific mutations or biomarkers found in their cancer without regard to the anatomical location of the tumor or its histology.), where patients are assigned to receive treatment based on the genetic changes found in their tumor [18,19]. The trial has 39 treatment arms evaluating 143 genes [20]. This trial aimed to investigate less common tumors by enrolling at least 25% of patients with diagnoses of rare malignancies. Currently, 62.5% of enrolled patients have tumors other than the four most common cancers (breast, colorectal, non-small cell lung, and prostate) [18]. Another clinical trial assessing the efficacy and safety of targeted therapies in patients who have exhausted standard treatments is the Targeted Agent and Profiling Utilization Registry (TAPUR) trial, which has 117 enrollment sites [21]. The goal of the TAPUR trial is to identify molecular signals predictive of treatment activity for drugs approved in different tumor types that share a similar genomic profile. Additionally, the TAPUR trial was designed to act as a vehicle to educate providers about precision oncology [22].

The NCI-MATCH and TAPUR trials are not only expanding the reach of precision genomics, but their results are also anticipated to provide tremendous amounts of new genomic information to the oncology community. Despite the advances in genetic technology, there are challenges to translating the results of genetic sequencing into patient care. This calls for the need of an enterprise consisting of multidisciplinary teams equipped with clinical and scientific tools to help prepare the oncology community to integrate precision medicine into clinical practice.

Current models of the precision oncology practice include specialized clinics, consultation services, or molecular tumor boards (MTB) [11,23,24,25,26]. MTBs consist of an interprofessional team who interprets NGS results and analyzes tumor cases at the molecular level to guide patient-specific targeted therapy [27]. A report from 2012 describes the MTB experience at the University of California San Diego Moores Cancer Center that was established to provide genomic profiling services to patients with advanced breast cancer. The MTB at this institution included physicians, scientists, geneticists, and bioinformatics. They found that among the 43 patients who participated, 40 (93%; mean age, 59 years) had at least one actionable mutation and 17 (40%) of these patients were treated with recommendations from MTB. Among the patients who were treated, seven (16%) achieved partial response or had stable disease for four or more months. This report demonstrated that MTB can optimize the management of patients with advanced, heavily treated breast cancer [28].

The pharmacist’s role in precision medicine practice models includes but is not limited to interpreting tumor genome sequencing results, managing anticoagulant use around the timing of tissue biopsy, leading patient education, and obtaining off-label drug therapy [11]. However, the pharmacist’s role may vary depending on each practice setting. This article describes the role of pharmacists in the precision oncology initiative at the Indiana University Health Simon Cancer Center (IUSCC).

## 2. Indiana University Health-Simon Cancer Center (IUSCC)

The IUSCC precision genomics program (PGP) was established in 2014 by a multidisciplinary team, including a medical oncologist, a medical and molecular genetic scientist, and an oncology pharmacy specialist. The program now includes a diverse team of medical oncologists, scientists, an oncology pharmacy specialist, two nurse coordinators, two genomic counselors, a data manager, two rotating PGY-2 oncology pharmacy residents, and an oncology fellow [29]. The PGP receives approximately 15 to 20 referrals per week. Since 2014, over 1000 patients have been sequenced in this program, with 83% of these patients receiving genome-directed recommendations and proceeding to either new treatments or clinical trials [29].

**Figure 1:**
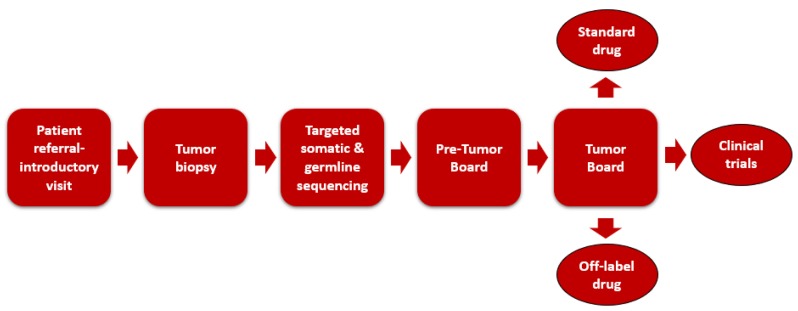
Oncology practice model at Indiana University Simon Cancer Center.

Patients with advanced or rare malignancies are referred to the program by their primary oncologists from both within IU Health and external health systems. A dedicated nurse coordinator schedules patients for an initial visit with the PGP oncologist to introduce patients to the program, obtain past medical history, and complete consent for biopsy. The PGP oncologist specifies the biopsy site, and a laboratory personnel conducts tumor sequencing on the biopsy specimen. The biopsy of the tumor can be performed at the initial visit, or tumor samples from other recent biopsy procedures can be sent to Foundation Medicine for NGS. Before the biopsy is scheduled, the oncology pharmacist reviews patient medications to identify those that may increase the risk of bleeding or impair wound healing. These medications include oral and intravenous chemotherapy agents, such as vascular endothelial growth factor (VEGF) inhibitors, and anticoagulants. Recommendations are made by the pharmacist to hold the high-risk medications for a specified amount of time before proceeding with the biopsy. Treatment options are proposed based on the sequencing results and an extensive literature review during a weekly pre-MTB meeting. The weekly MTB conference consists of medical oncologists, oncology pharmacists, oncology nurses, pathologists, genetic counselors, residents, fellows, and a phase I clinical trials team. The past medical history and tumor sequencing results of each case are presented by either the medical oncologist, genetic scientist, or oncology pharmacist, followed by a discussion of genomic targets and potential therapies. Decisions regarding therapy are given with considerations to patient-specific factors (e.g., comorbidities, performance status, prior drug exposure, etc.) and genomic factors (e.g., strength of genomic association, inherited genetic variants that would predict increased risk from a drug, etc.). To standardize targeted therapy recommendations at IUSCC PGP, identified targets are chosen, with first priority given to DNA mutations, copy number variations, and DNA fusions; second priority to mutations detected by immunohistochemistry or by florescence in situ hybridization; third priority to mRNA overexpression alone. The oncology pharmacist documents the recommendations from the tumor board conference into the EMR. An overview of the precision oncology practice model at IUSCC is shown in Figure 1. 

After the results are finalized, the patient has a return clinic visit with the medical oncologist, scientist, and pharmacist to discuss the genomic results, molecular biology of genomic aberrations, potential therapeutic options, and if appropriate, clinical trial referral options. A genetic counselor sees all patients with clinically relevant germline mutations that are detected from the tumor biopsy. The oncology clinical pharmacist and PGY-2 oncology pharmacy residents will meet with each of the returning patients for 30 to 45 min to educate them regarding the genomic findings, treatment recommendations, medication details (dose, side effects, administration), and clinical trial information. It is crucial to assess patients’ health literacy before each counseling session and use effective communication skills, such as active listening, using patient-friendly language, and assessing patient understanding. Each counseling session will begin by introducing the patient to what will be discussed during the session: a brief overview of genetics, a review of the precision medicine program and the process of how the treatment recommendations are made. Visual demonstrations such as simple drawings of cancer genetics pathways as well as easy-to-understand analogies are utilized when explaining the molecular biology of genetic mutations and the mechanism of action of treatment. Described below is an example of the education session that was conducted for a 61-year-old male with BRAF-mutated metastatic colon cancer who was seen at IUSCC PGP clinic.

Pharmacist:

“We are going to start with briefly introducing you to what we do here at the Precision Genomics clinic, explain the process of how we came up with the treatment recommendation based on the sequencing results, and go over the recommended treatment with you. At this clinic, we view cancer at a genetic level. We took a biopsy of your tumor, sent a sample to a lab that utilizes technology to identify if there are genetic changes in your tumor that we can target with medications. After we obtain the results from your biopsy, we present the findings to a multidisciplinary tumor board, which consists of 15 to 20 individuals including oncologists, pathologists, nurses, pharmacists, and scientists to discuss and devise treatment specific to you. What questions do you have so far?”

Pharmacist:

“As you see here in this drawing, every cell has a DNA, which is the blueprint of life as it contains information needed for individuals to grow and survive. Cells must divide in order for us to survive but there must be a balance between cell division and cell death. As we age and with increased exposure to environmental factors and genetic changes, we may acquire certain mutations or “mistakes” in our genes that can lead to the development of cancer. From your tumor biopsy, we found something called a BRAF mutation. BRAF is a gene that tells the DNA to make a protein called B-Raf, which is involved in a pathway called the MAPK pathway that is responsible for controlling how quickly your cells divide, and it acts as a gas pedal that tells your cell to grow and multiply (the pharmacist will use a drawing to show this pathway). Normally, BRAF has an off-switch that controls when the cells should divide. However, when BRAF is mutated, the growth signal will always be on causing cancer cells to grow and divide at a much faster rate than normal.” After answering patient’s questions, the pharmacist proceeds with explaining in detail the recommended treatment including mechanism of action, dose, administration, side effects, drug-interactions, etc.

The genomic findings and treatment recommendations are also summarized in writing and given to the patient along with PGP contact information, drug information, and if available, information on the recommended clinical trials. The summary of each visit is documented in the EMR by PGY-2 oncology pharmacy residents and communicated to the referring primary oncologist. The subsequent patient interactions related to clinical trials consultation and drug procurement, including letters of medical necessity, prior authorizations, and peer to peer discussions are completed by the oncology clinical pharmacist or PGY-2 oncology pharmacy residents. 

In 2016, the precision genomics team including the clinical oncology pharmacist at IUSCC led a formal prospective cohort study evaluating the efficacy of a precision medicine approach, using genome-guided therapy designed by IUSCC multidisciplinary MTB team members. In this study, 101 patients with metastatic solid tumors who progressed on at least one line of standard of care therapy were evaluated from April 2014 to October 2015. The median number of prior regimens was four in both groups. The most common malignancy diagnoses in the genomically vs. non-genomically-directed therapy groups were soft tissue sarcoma (22.7% vs. 19.3%), breast (18.2% vs. 14%), and colorectal cancer (15.9% vs. 8.8%). Of 44 patients treated with genomically guided therapy, 19 (43.2%) patients attained a progression free survival ratio ≥ 1.3 vs. three of 57 (5.3%) patients treated with non-genomically guided therapy (*p* < 0.0001). Furthermore, patients treated with genomically guided therapy had a superior median PFS compared to those treated with non-genomically guided therapy (86 days vs. 49 days, *p* = 0.005, HR = 0.55, 95% CI: 0.37–0.84) [30]. 

## 3. Clinical Pharmacist Role at IUSCC Precision Genomics Clinic

At IUSCC, the hallmark feature demonstrating the successful experience of transforming precision oncology from a scientific concept to clinical practice was the interprofessional dynamic and strong team-based approach. Particularly, the oncology pharmacy clinical specialist has a leading role throughout the continuum of precision genomics and is heavily relied upon by physicians and nursing colleagues. Key responsibilities of the oncology clinical pharmacist at IUSCC PGP include collaborating with oncologists and scientists to interpret genetic sequencing and provide recommendations, documenting clinical notes in the medical record, educating patients, facilitating off-label drug procurement, and ensuring patient safety when obtaining tissue biopsies.

Interpretation of genetic sequencing and provision of treatment recommendations require scientific knowledge of oncogene pathways, tumor suppressor genes, the clinical significance of genetic variance, treatment toxicities, etc. Clinical and scientific proficiency in this area may be achieved via certificate-based training programs and extensive self-directed learning, as well as knowledge of clinical trials, drugs in development, and existing targeted therapies.

As experts in pharmacotherapy and oncology treatment, the clinical pharmacists at IUSCC PGP lead the management of anticoagulation and other interacting therapies, to ensure patient safety prior to obtaining tissue biopsy for genomic testing. Timing of anticoagulation and VEGF inhibitors administration are only a few examples of therapy interventions that clinical pharmacists manage at IUSCC.

The PGP at IUSCC serves as a one-stop-shop that provides comprehensive services, which greatly impacts patient access to targeted therapies and decreases delay in treatment initiation. This is another area where the oncology clinical specialist plays a leadership role within the genomics program. Being experts in pharmacotherapy, clinical pharmacists when compared to physicians or other allied providers are better equipped to take ownership of the process of obtaining medications, communicating with insurance companies on behalf of physicians, and navigating patient assistance programs. When oral targeted therapy is recommended, a prescription is sent to IUSCC specialty pharmacy. The specialty pharmacy team then submits prescription claims to the patient’s insurance. Outside of clinical trials, the recommended targeted therapy can be off-label, which is often denied by insurance companies. If prescription coverage is denied, the PGP clinical pharmacist sends an appeal letter and performs peer to peer discussions after gathering extensive literature to support the recommendation. If multiple attempts are denied, the clinical pharmacist seeks additional financial resources, such as applying for pharmaceutical manufacturer patient assistance programs. While the process of off-label drug procurement can be time consuming, it is an essential step to ensure patient access to genome-directed therapy.

Finally, selection of a particular practice infrastructure may be influenced by the institution’s payment model. Financial sustainability is key when implementing a novel service such as precision oncology. With a healthcare payment model dominated by fee-for-service in the United States, an ambulatory clinic model that bills for individual clinic visits and laboratory tests can be viewed as revenue generators. In contrast, consultation services and MTBs are more likely to be viewed as cost centers whose services might need to be justified. Regardless of what avenue is pursued when implementing precision genomics in oncology practice, oncology pharmacists hold essential clinical and leadership roles in many aspects of precision oncology practice.

## 4. Challenges and Opportunities

The number of clinically relevant, druggable genetic changes and approved targeted therapy are anticipated to increase at an exponential rate, given advancement in genomic testing technologies and efficient clinical trial designs. This rapid shift in practice from cytotoxic therapy to targeted medicine poses challenges yet brings exciting opportunities to pharmacists practicing in hematology and oncology.

Incorporation of genomic testing and targeted therapy is considered standard of care in many solid tumor malignancies including lung, colorectal, and gastrointestinal [11]. While the role of certain targeted therapy is well established in specific tumor types, such as the use of ALK inhibitors in ALK-rearranged non-small cell adenocarcinoma, the efficacy of tissue-agnostic therapy when used off-label and off-clinical trials may not be well characterized [31]. Patients presenting with multiple targetable genetic changes can make therapy selection especially challenging. As a result, an interprofessional, MTB-based approach can offer scientific and clinical analysis of genomic testing results to recommend treatments specific to each individual patient. The market of targeted therapy is expected to continue to grow following the results of the NCI-MATCH and TAPUR trials, leading to an increase in demand for structured, MTB-based precision oncology services. As described above, pharmacists play a leadership and an integral clinical role in the practice of precision oncology. When equipped with adequate training or equivalent experiences, clinical oncology pharmacists have exciting opportunities to collaborate with other providers to establish and lead precision genomics services.

The practice of precision oncology may not be a new concept anymore, but it is still considered a novel approach to patient care in oncology. The IUSCC PGP demonstrated clinical benefit in using precision genomics guided therapy in advanced and refractory solid tumors. These encouraging findings indicate an opportunity for advancing the practice of precision oncology via research. Examples of areas for future research include efficacy of off-label, histology-independent treatment, rare patient cases with successful outcomes associated with targeted therapy, impact of precision genomic programs on improving patient access to targeted therapy, and expansion to multicenter studies.

To successfully implement precision genomics services and conduct research, pharmacists need to be equipped with adequate knowledge and proficiency in interpreting genomic testing and targeted therapy selection. This reveals gaps in pharmacy basic and higher-level education [32]. In the IUSCC practice model described above, pharmacists are heavily involved in interpreting genomic tests and making clinical recommendations. In fact, pharmacists are principal investigators of associated research projects and co-leaders working alongside medical oncologists and scientists. The ongoing advancement in genomic testing, clinical trial design, and precision medicine call for a long overdue transformation in pharmacy didactic curriculum and experiential education to reflect the practice-changing evolution in precision medicine and genomic-directed therapy.

Opportunities to bridge current gaps in pharmacy education include offering PGY-2 oncology pharmacy residents practice exposure to facilitate understanding of molecular biology, genomic signaling pathways associated with tumorigenesis, genomic sequencing technology, and targeted therapy selection. Understanding that an on-site experience may not be possible, other avenues for experiential learning include off-site rotations, webinars, case discussions, MTB meetings organized by the American Society of Clinical Oncology, etc. Another approach that can potentially reach a larger population of learners include a virtual learning experience led by experts in the field, consisting of a month-long rotation with various activities such as topic discussions, patient cases, and journal presentations.

## 5. Conclusions

The practice of precision oncology utilizing an MTB-based model is an emerging approach transforming precision genomics from a novel concept into clinical practice. Only a few precision genomics programs in the United States have a strong pharmacy presence with clinical pharmacy specialists serving leadership roles in research, interpreting genomic testing, and making treatment recommendations. Recent advancements in precision oncology have revealed gaps in pharmacy didactic curriculum and experiential education in precision medicine and genomics. Pharmacists in clinical practice and academia have a professional obligation to transform the education system and to prepare trainees for opportunities in precision medicine and contemporary practice.
